# Implementation of Thermal Camera for Non-Contact Physiological Measurement: A Systematic Review

**DOI:** 10.3390/s21237777

**Published:** 2021-11-23

**Authors:** Martin Clinton Tosima Manullang, Yuan-Hsiang Lin, Sheng-Jie Lai, Nai-Kuan Chou

**Affiliations:** 1 Department of Electronic and Computer Engineering, National Taiwan University of Science and Technology, Taipei 10607, Taiwan; D10902809@mail.ntust.edu.tw (M.C.T.M.); m10902137@ntust.edu.tw (S.-J.L.); 2 Department of Informatics, Institut Teknologi Sumatera, South Lampung Regency 35365, Indonesia; 3 Department of Cardiovascular Surgery, National Taiwan University Hospital, Taipei 10002, Taiwan

**Keywords:** thermal camera, contactless sensors, non-contact, physiological measurement

## Abstract

Non-contact physiological measurements based on image sensors have developed rapidly in recent years. Among them, thermal cameras have the advantage of measuring temperature in the environment without light and have potential to develop physiological measurement applications. Various studies have used thermal camera to measure the physiological signals such as respiratory rate, heart rate, and body temperature. In this paper, we provided a general overview of the existing studies by examining the physiological signals of measurement, the used platforms, the thermal camera models and specifications, the use of camera fusion, the image and signal processing step (including the algorithms and tools used), and the performance evaluation. The advantages and challenges of thermal camera-based physiological measurement were also discussed. Several suggestions and prospects such as healthcare applications, machine learning, multi-parameter, and image fusion, have been proposed to improve the physiological measurement of thermal camera in the future.

## 1. Introduction

### 1.1. Research Motivation

The use of thermal cameras has become very widespread in recent years as it can be applied in various fields. Thermal cameras have the advantages of operating in an environment without light and not being affected by changes in light. There are existing studies illustrating that thermal camera can be used to monitor respiratory rate (RR), heart rate (HR), and body temperature, while other studies found its use in breast cancer diagnosis [1], evaluating physical condition [2], stress level [3], as well as the neonates’ health condition [4], sleep posture [5], and many more not mentioned here.

Meanwhile, vital signs data, such as blood pressure, temperature, respiration rate, and heart rate, are critical for patient care and diagnosis. They enable physicians and other healthcare workers to make informed decisions about a patient’s treatment options and overall well-being. However, existing medical instruments still rely on physical touch from existing tools to gather data about patients’ health. Most techniques for determining respiration and heart rate include physical contact with the patients such as pulse oximeters, ECG (electrocardiogram), monitoring systems using electrodes, or piezoelectric sensors.

During the SARS-CoV-2 19 pandemic, the whole world reduced the amount of direct contact drastically. People are reluctant to visit health and medical institutions due to fear of infection. Based on existing studies [6], there has been a change in medical services since the outbreak of SARS-CoV-2 19. Contactless services have been implemented during SARS-CoV-2 19, and will become commonplace [7] even after the pandemic. Several developments such as measuring RR and HR using the non-contact method with radar sensors [8], blood volume pulse and vasomotion measurements [9], using radio frequency, and the Doppler effect to monitor vital body objects [10] have been tested and researched.

Body temperature is an excellent indication of a patient’s health [11]. Human body temperature can be categorized into two types: skin temperature and core body temperature. Skin temperature is the temperature of the outermost surface of the body. Average human skin temperature varies between 33.5 and 36.9 °C (92.3 and 98.4 °F) while healthy core body temperature falls within 37 °C (98 °F) and 37.8 °C (100 °F) [12]. According to a study [13], extreme body temperature can negatively affect how a human’s body and vital organs work. To compensate for this, the body has thermoregulation processes that enable it to maintain a standard core internal temperature.

Non-contact systems can detect temperature through infrared thermography because it can detect electromagnetic waves produced by anything with a temperature greater than absolute zero Kelvin. This phenomenon is used for the development of thermal cameras. Thermal cameras measure temperature using infrared radiation from 1 to 14 µm spectral range [14,15]. This measurement procedure is known as infrared thermography (IRT). IRT is a non-invasive technique that remotely measures the energy emitted by an entity (i.e., human body, industrial machine, engine, and many more objects). IRT is applied as an indirect technique for capturing the changes in surface body temperature and can be used to measure other physiological signals [16]. However, the commonly used thermal camera for medical purpose is a long-wavelength infrared (LWIR) type with a 7–14 µm spectral range [17].

According to research in United States by McKinsey between March and April 2020, a large migration to telemedicine occurred, coinciding with an over 80% drop in in-person visits. The use of telemedicine by physicians and healthcare organizations has also expanded by 50–175 times since the COVID-19 outbreak [18]. The increase in public demand for indirect healthcare during the pandemic led to the rapid development of non-contact healthcare practice and emphasized its importance. There is also a trend for non-contact measurement technology to replace the current conventional methods without any compromise on performance and accuracy. However, the use of thermal cameras to capture vital signs has its challenges in terms of image and signal processing. 

### 1.2. Research Objective

This paper aims to systematically evaluate the use and development of thermal cameras in its application for measuring vital signs using preferred reporting items for systematic reviews and meta-analyses (PRISMA) to produce relevant papers from 2012 to 2021. More specifically, this paper will evaluate system capabilities, thermal camera types, signal processing steps, system platform, and highlight system performance along with the validation method. This systematic review contributes an evaluation of recent progress in non-contact physiological measurement with LWIR thermal camera that can be used as a basis for reference for other related research.

### 1.3. Comparison with Existing Reviews

Other previous systematic review papers that also discussed the applications of thermal cameras are listed in Table 1.

There are some significant differences between this systematic review and the existing ones. These differences mean that this study has a novelty in review. The following studies [19,20] have similarities with this study regarding research objectives, but in these the coverage years are different. Some studies also have a scope that focuses on specific aspects such as sports [21], psychophysiological [22], breast cancer [1], neonatal [4], vein finder [24], and human core temperature [26]. Some studies have a broader scope; for example, the study conducted by He et al. [27] covered all aspects outside the health field.

## 2. System Architecture in General

The image processing carried out by each study requires a platform on which the processing takes place. Most of the studies carried out signal processing on software such as python for a programming language and OpenCV as a library framework. Python is a multi-hardware programming language that runs on various hardware ranging from personal computers to mini-PC boards such as Jetson [28] and Raspberry Pi. OpenCV stands for Open-Source Computer Vision, a library that provides various kinds of image processing functions which can be used for real-time processing directly from the camera or by using pre-recorded image data.

**Figure 1:**
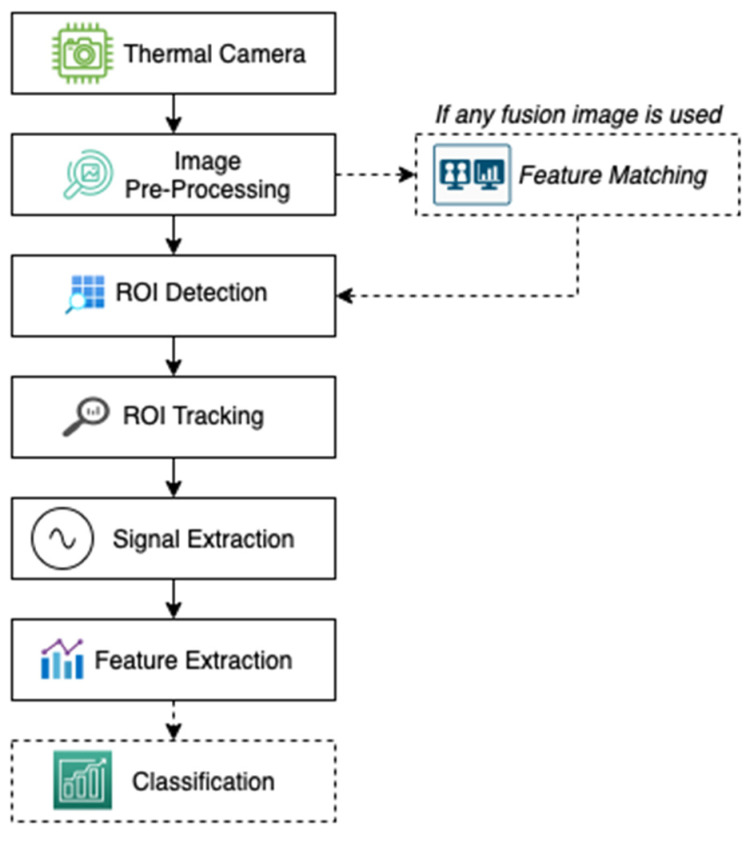
The general process stages of studies using a thermal camera that performs physiological measurements. Several stages are depicted by dotted line boxes explaining that these stages only apply in certain studies in general.

Although each study that uses a thermal camera for physiological measurement included in this systematic review is very diverse in various vital signs, the processing stages of each study can generally be summed up in one process flow that can be seen in Figure 1.

### 2.1. Thermal Camera Model and Specification

The image process stage begins with the acquisition of a thermal image from a thermal camera. The resolution and the number of images that run in one second or what is known as frames per second (FPS) are essential in signal processing, primarily related to images. Several cameras are used more than once by the studies included in this systematic review, i.e., the A315 and A325 from Teledyne FLIR LLC and the MAG62 from Magnity Electronics Co., Ltd., Shanghai, China.

There are several necessary specifications related to thermal cameras. In general, the cameras used in the included studies can record video from the lowest FPS of 8.7 FPS to 60 FPS in resolution between 160 × 120 pixels to 1024 × 768 pixels. FPS and resolution are closely related to the quality of the resulting signal [29,30]. FPS refers to the number of thermal image frames captured in one second. The more image frames obtained, the greater the variation of thermal information and its variability. Therefore, in this case, FPS can be interpreted as the sampling rate of a system. Likewise, the image’s dimensions also show the number of measurement points made by the thermal camera. The larger the image dimensions, the easier it will be for the system to detect region of interest (ROI). 

Apart from thermal image specifications, two variables are often shown regarding the performance of thermal cameras, i.e., temperature accuracy and thermal sensitivity. Temperature accuracy indicates how close a measurement from a thermal imager is to the actual absolute value. Meanwhile, thermal sensitivity refers to the noise equivalent temperature difference (NETD). This value specifies the most negligible temperature difference that the camera can detect.

Some research shows that NETD is a critical aspect to show the performance of a thermal camera [31,32,33,34]. The value of NETD is also an essential variable in using low-cost thermal cameras for applications in the medical field. For example, a thermal camera with a NETD value of less than 50 mK is ideal for medical applications [35]. In studies related to the measurement of respiratory rate and heart rate, the value of the NETD becomes an essential aspect because the measurement of respiratory rate considers changes in temperature rather than the value of the temperature itself. Meanwhile, the study conducted by Pan et al. [36] made corrections to the value of body temperature readings with correction variables in measuring body temperature, while a study conducted by Rao et al. [37] developed an automatic temperature correction algorithm to calibrate the camera according to black body reference.

The correction value from the thermal camera is obtained by calibrating it. This calibration process is performed using a radiometric calibration method, which establishes the relationship between the pixel signal and the temperature of the target object [38,39]. Calibrated thermal cameras minimize temperature readings that differ significantly from reference devices and become more reliable for medical applications.

More complete details regarding the use of thermal cameras in each study are summarized in Table 2.

### 2.2. Image Pre-Processing and Feature Matching

The second stage is the pre-processing of the thermal image. Pre-processing is carried out on the entire frame of the image. During pre-processing, gaussian filter, changes in image dimensions or size, conversion of the number of FPS, altering color channels to grayscale, bitmap, or using pseudocolor can be applied.

**Figure 2:**
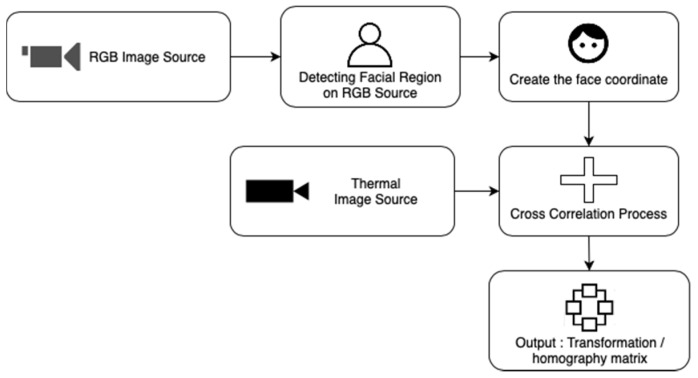
An overview of how RGB cameras are used to assist thermal cameras in determining ROI and the transformation process.

A feature matching stage is required for studies that use more than one camera or what is known as image fusion. The fusion cameras used are also varied. Some use a combination of near-infrared (NIR) and LWIR cameras [40,53], while the others use a combination of LWIR and RGB cameras [42,47,48,52]. Most studies combined the two images, generally thermal images and RGB images. The RGB image is used for ROI detection. The ROI coordinates in the RGB image are matched with the coordinates on the thermal camera. Determination of these coordinates has a diverse method, ranging from multispectral localization using the dlib algorithm [55,56], and pre-trained machine learning models. These points are then correlated with the thermal image. Some pre-processing may be required, such as frame per second synchronization and adjustment of the image’s dimensions (in general, the dimensions of thermal images are often smaller than RGB images). This cross-correlation process also has several algorithms, including affine transformation [57], the Oriented Fast and Rotated Brief feature [58,59], and others. This cross-correlation process will produce an equation matrix called a homography matrix (some studies call it a transformation matrix or correlation matrix). An illustrative simplification of this process can be seen in Figure 2.

### 2.3. Determining and Tracking of ROI

Determining the ROI in a dimensional image is an important stage in thermal camera image processing. There are several methods used by the studies selected in this systematic review. The first one that was used is called Viola-Jones framework [60]. Paul Viola and Michael Jones developed this framework using the haar feature, commonly used to detect facial parts. All studies by Negishi [42,47,48] included in this systematic review used Viola-Jones to determine ROI. However, Hu et al. [51] claimed that their ROI detection algorithm has better performance when compared to Viola-Jones, with 98.46% accuracy versus 87.69%. Chen et al. [52] also argue that Viola-Jones in OpenCV is always used to determine coarse faces’ locations but is not precise in respiratory rate measurement. Therefore, deep learning is used as a method to determine ROI.

Movement between frames, especially when the camera is set at a low FPS, is often a problem in signal acquisition from the thermal camera. For this reason, optical flow is used to solve this problem. The study conducted by Lyra et al. [28] used optical flow to quantify the thermal image so that the subtle motion in the chest area is reduced for later extraction of the respiratory signal. Furthermore, the study conducted by Scebba et al. [40] used a dense optical flow algorithm developed by Farneback to reduce the periodic motion of the torso.

Extracting signals from moving objects is a challenge, and therefore tracking methods are needed to overcome them, one of which is by using The Kanade-Lucas-Tomasi (KLT) algorithm, which is used by several studies [40,51,52]. This algorithm uses a linear coordinate mapping which determine the corresponding region in the thermal video. This tracker extracts feature points from ROI using the minimum eigenvalue algorithm and follows those points with a single point tracker.

### 2.4. Signal Extraction, Feature Extraction, and Classification

Signal extraction is an advanced stage that is carried out when the system has succeeded in identifying ROI and tracking ROI movements. Vital physiological signals are plotted in units of time known as time-series signals. The general method for extracting this signal is by comparing pixel-per-pixel motion in thermal images [61]. Not infrequently, the extracted signal requires post-processing in a filter until the signal results can characterize a change (signature). These changes contain data that must be extracted at the feature extraction stage. Some algorithms can be used for extracting the feature, i.e., peak detection [62,63], fuzzy rule [64], one dimensional CNN [65], power spectral density [66], and various other methods. There is also a python toolkit for quickly extracting the feature [67] in a python package library. 

The results of this feature extraction will show a value that can be drawn into the system’s output. In some studies, this output cannot be easily interpreted. Using machine learning or deep learning [43,45], a classification of the signals generated by the previous stages is carried out based on the model trained beforehand. This output is a form of classification that users can easily interpret. Each input will be directed to two or more outputs, either anomaly detection or multi-class output, using classification and machine learning.

## 3. Thermal Camera for Physiological Measurement

This systematic review reviews the use of thermal cameras for physiological measurements which will be broken down into three subsections, including respiratory rate, heart rate, and body temperature.

### 3.1. Respiratory Rate

#### 3.1.1. Overview of Respiratory Rate Measurement

Monitoring RR and related variations are critical for determining an individual’s health status [68]. Moreover, research [69] states that the RR can be used as one of the most efficient indicators to determine whether a person is healthy. Anomalous RR is critical for detecting significant health problems and can also be used to forecast potentially serious clinical outcomes such as influenza classification [42], lung airflow limitation [70], and sleep apnea screening. Additionally, monitoring variations in RR can assist in identifying a high-risk intensive care patient up to 24-h before a medical emergency.

RR is defined clinically as the times of respiration recorded within a minute (in breaths per minute, or bpm). In general, the RR is normal if it is 12–20 bpm for adult humans. A study [71] shows that a slight increase of breaths per minute to 24–28 correlates to an increased risk of mortality by 5%. During the COVID-19 pandemic, RR counting is essential. RR is also a vital sign that determines the severity of SARS-CoV-2 19 infection [72]. This viral outbreak has caused many ICUs to be at full capacity and high bed occupancy rates throughout the world. Medical equipment, including instruments for measuring vital signs, is insufficient in some countries [73]. The thermal camera is undoubtedly one potential solution for developing a RR counter that works without direct contact.

While RR is a significant clinical predictor of severe events, it is often measured manually, yielding erroneous findings. RR was often not regularly recorded, even when the patient’s main complaint was a respiratory illness [68].

There are several well-known methods used for RR monitoring [68]. The first is by using a manual human counting method. However, it might be inaccurate and time-consuming. The second is using a spirometer. This method is considered accurate and also measures some other respiratory parameters. However, it can interfere with natural breathing and difficult for continuous RR monitoring. The third approach employs capnometry, a highly accurate, simple, and measured continuous monitoring technique. This is a contact approach that is not particularly pleasant and requires analysis using specialized equipment. The last approach is impedance pneumography, which is precise, continuous, and concurrent. However, this procedure is challenging to conduct and requires specialized tools for analysis.

In addition to standard medical measurements used as a reference in hospitals, there are also several affordable, contact-based methods to measure RR, such as using a nasal temperature probe near the nostril [74] or using a microphone located near the nostril that records the inhale-exhale sound noise [75]. The primary drawbacks of these methods stem from their intrusive nature: they may be unpleasant and potentially disruptive to sleep which may alter the results. Additionally, patient movement and any other signal noise may dislodge the sensors or skew the data.

Noninvasive methods for detecting breathing include non-contact audio analysis, vibration sensors, thermal imaging, and doppler radar sensors. The extraction of breathing sounds from sensor data polluted by ambient noise is significant for non-contact audio analysis. Vibration sensors impose positional and postural limitations and need the use of costly specialized hardware. By detecting the breath as it is exhaled, thermal imaging methods were utilized to record a breathing signal. Thermal imaging can be achieved using a thermal camera. One of the advantages of a thermal camera is that it can be used indirectly and reliably without affecting the light intensity, and can be used in a completely dark room. In general, the challenge in using a thermal camera is processing thermal images and extracting features from these images.

#### 3.1.2. Summary of Thermal Camera Usage Related to Respiratory

Next, Table 3 summarizes all the characteristics of the studies with respiration as the main objective that were used in this systematic review.

#### 3.1.3. Deep Learning for RR Monitoring

Several studies use deep learning to classify the breathing pattern and to determine the ROI from the image. There are some deep learning algorithms used in the research list above, CSPDarknet [28], FlowNet 2.0 [76] (algorithm based on deep networks), k-nearest neighbors (k-NN) [43,45], and cascade convolutional neural network (CCNN) [40].

CSPDarknet is the backbone running on YOLOv4. Lyra et al., in their research [28], used it to determine four classes that would be used as ROI, namely head, chest, patient, and clinician. Head ROI is used to measure body temperature, chest ROI is used for RR estimation, and clinician ROI is used to count the number of clinicians near the patient. After the chest ROI was determined, respiratory movement was tracked using a pixel-wise temporal mean algorithm by comparing movement between frames. The use of the neural network to determine five facial landmarks were also used by Scebba et al. [40] by utilizing CCNN on NIR images.

Meanwhile, Jagadev et al., in both of their studies [43,45], used k-NN to decide whether the human volunteer had normal or abnormal respiration. Previously, the breath detection algorithm (BDA), which they also developed, was used to extract respiratory movements. In simple terms, BDA calculates the number of peaks and valleys based on the specified ROI movement from the nostrils. Finally, the output of this BDA is forwarded to the k-NN to classify between normal breathing, bradypnea, or tachypnea (abnormal). The output of this system is compared with the Support-Vector Machine (SVM) method to determine the accuracy achieved.

#### 3.1.4. Camera Sensor Fusion: Usability and Image Fusion Method

Several studies on the list combined the two types of cameras with different measurements related to respiratory. Most of them use a combination of a LWIR thermal camera with a CMOS RGB camera or a color camera that we commonly find on smartphones, webcams, or point-and-shoot cameras. The merging of these two cameras aims to gain the advantages of each camera and eliminate the weaknesses of each camera. Meanwhile, light significantly affects RGB cameras, and this type of camera cannot be used in low or no light conditions. In contrast, thermal cameras can capture objects even in light conditions because they work using the principle of radiation emitted by objects. Table 4 summarizes each study involving fusion cameras and their characteristics.

The following spectral video fusion study [40] combines two types of thermal cameras, which are NIR and LWIR cameras. Scebba et al. initiated a new algorithm to calculate the RR based on multispectral data fusion from the two cameras. The multispectral ROI localization analyzes footage from LWIR and NIR cameras. The localized ROIs extract the Thermal Airflow (TA) signal from the nose ROI and the respiratory motion signal from the chest ROIs in the LWIR and NIR cameras. The RR and signal to noise ratio (SNR) are calculated in the Signal Quality-based fusion (SQb Fusion) using the TA’s frequency analysis and respiratory motion signal both from the LWIR camera and the NIR camera. The weighted median generates an RR estimation by combining all RR estimations and weighting them by their SNR. Temporal aspects and frequency characteristics of TA, respiratory motion from NIR and LWIR cameras are used as input to the ensemble of support-vector-machine to determine whether apnea occurs or not. The intelligent signal quality-based fusion (S^2^Fusion) algorithm combines the findings of the SQb Fusion with the apnea classifier (h) to produce an apnea-sensitive signal.

There is also the use of two different cameras to measure two different physiological quantities. Negishi et al. used the same two cameras configuration in all three studies [42,47,48]. LWIR camera is used to measure RR while RGB camera is used to measure HR. Image fusion determines ROI based on RGB images rather than thermal images. To determine the ROI of the nose and mouth, a feature matching analysis was performed with a homography matrix between RGB and thermal images based on the contours of the human face. Grabcut is used for facial contour extraction, while oriented-fast and rotated brief (ORB) algorithm is used for feature matching and dlib for determining the region of nose and mouth. All these algorithms and tools are available as libraries in OpenCV.

Almost the same as before, Hu et al., in their studies [51], also use camera fusion to make the determination of ROI while facial objects was detected using the RGB camera. To record thermal and visual images, an affine transformation is needed. The first step is to pick the most correlated points in the first frame of bimodal movies to determine the thermal image’s fixed point and RGB image’s image points. Following that, cross-correlation is used to modify these points in order to produce the transformation matrix. After mapping between the RGB image and the thermal image, the bounding box is determined for the ROI object (the face, nose, and mouth) by using the Viola-Jones algorithm. The shi-Tomasi corner detection algorithm is used to help extract the interest points to calculate the covariance matrix, while the KLT algorithm is used to track ROI on movement.

As before, the use of RGB camera for face detection is also used by Chen et al. [52]. They provide an alternative method to measuring RR if no face is detected by tracking the sticky markers that placed on the body. Meanwhile, to combine RGB and thermal images, they use affine transformation to transform two different geometric shapes. The Viola-Jones algorithm is used to detect faces, while the KLT algorithm is used for tracking the ROI.

#### 3.1.5. RR Signal Extraction Process

**Figure 3:**
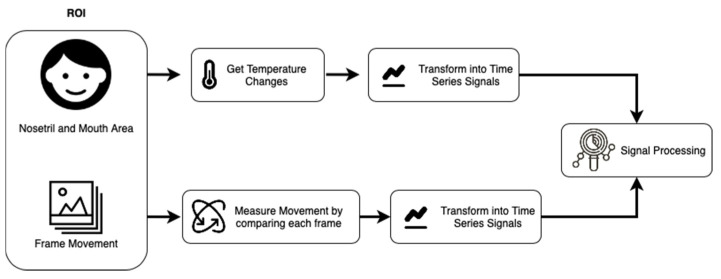
An overview of how the signal extraction process from a thermal image is carried out. In general, there are two methods: first by measuring changes in temperature in the area around the nostrils and mouth, and second by looking at the movement based on the comparison between changes in pixels in each frame.

A thermal image only has a single information channel that is a temperature representation converted into an image matrix. It is by utilizing this only information that a respiratory signal can be generated. In general, as shown in Figure 3, two changes can be observed: the first is a change in the temperature value and the second is a change in movement. Each of these characters will be explored by each study based on the methods and algorithms they use, respectively.

The study [44] conducted by Mutlu et al. used the temperature change around the nostril to indicate respiration. After defining the ROI and excluding non-varying pixels, the decreasing segments are identified using experimentally established criteria for a minimal frame-to-frame decline. If a single frame exists between two possible decrease segments, they are combined. The process of identifying temperature changes at the pixel level by comparing frame per frame was also used in other studies.

Another way of extracting the RR signal is to consider the movement of pixels between frames without making the nose or mouth the ROI. This method was used in the following study [54] and is more reliable when used in real time and with patients in the frame using a blanket or in a position not facing the camera. Breathing motion detection uses a subtraction technique in the background to identify motion by computing the difference between the current and previous frames. To be precise, the absolute difference between the current frame I(x,y,f) and the previous frame I(x,y,f−1) is computed for all pixels where x,y are the coordinates of x and y axis respectively, and f is the frame sequence. Then, employing thresholding, erosion, and dilation procedures, parts of the relocated region are removed. The parameters utilized in these procedures are 5 for thresholding, which ensures that the difference between the pixel values is smaller than 5, and 5 × 5 kernel for opening (i.e., erosion and dilation). Following that, boundary boxes are determined using contour detection and noise filtering. Finally, the RR is determined by the number of bounding boxes. The concept of comparing each pixel movement between frames per frame is also used by other studies [77].

Negishi et al., in three of their studies [42,47,48], use multiple signal classification (MUSIC) algorithms to calculate RR estimates. This algorithm is also proven to be more accurate than FFT in time series data with a shorter window. By using this algorithm, the correlation matrix from the time series data is calculated, and the eigenvectors are obtained.

#### 3.1.6. Performance Validation Method on RR

Testing on RR is carried out by comparing the system output with reference equipment such as apnea monitors, sleep diagnostic equipment, respiratory belts, and other respiratory rate measuring devices. For example, in this study [52], GY-6620 (South China Medical and Electrical Technology Co., Ltd., Zhengzhou, China) was used as a comparison. GY-6620 is the equipment used in polysomnography or sleep tests and it can provide output records of body activity during sleep, including RR. In another study [50], the SOMNOlab2 tool (Weinman GmbH, Hamburg, Germany) was used as the reference. The device is a measuring device for body activity that records thoracic movements based on piezo plethysmography. In his three studies [42,47,48], Negishi also used the same validation system, namely the respiratory effort belt. However, the model of the tool is only listed in one of their studies [48], namely DL-231 (S&ME, Tokyo, Japan). A similar force sensor-based respiratory belt was also used by another study [54], the Go Direct Respiration Belt model. This belt is set to record ten respiration samples per second for 5400 s. This study [44] also uses a respiratory belt to obtain the reference value. Unfortunately, not all studies compare the results with standard medical equipment; others use statistical calculations as a performance test method, such as scatter plots, bland-Altman plots, or other statistical calculations.

### 3.2. Heart Rate

Studies that use thermal cameras to measure heart rate tend to be less popular than those measuring respiratory rate. Many researchers use RGB cameras rather than thermal cameras for heart rate measurement. The heart rate measurement with the RGB camera utilizes changes in skin color that can be observed in one of the three color channels in the RGB image. While in thermal cameras, discriminant characteristics can be obtained based on the two most popular methods: using the blood perfusion temperature changes from a particular pixel [49,78,79] or by analyzing the head movement based on Balistocardiography (BCG) [80,81].

Similar to the respiratory rate process, obtaining a heart rate signal from a thermal camera begins with capturing the images, pre-processing the image, detecting the ROI, and tracking the ROI so the ROI will be more stable to movement.

Some studies describe the camera specification they use for the research. For example, Bennet et al. [79] use a FLIR-A camera with 640 × 480 pixels and 60 FPS framerate. On the other hand, Kim et al. [49] use a FLIR T430sc camera with 320 × 240 pixels and 12 FPS framerate. Unfortunately, Gault et al. [78] use pre-recorded thermal video with ten subjects without further detail about the camera specification.

For studies that are using temperature changes methods, they select some regions such as the highest blood vessel temperature region on the skin [49,78] or chest [79] as the ROI. In other parts, for the head movement-based method, they use the entire head as an ROI and track its movement [80,81].

Some studies reported that the noise was very high on the unprocessed signal and the discriminant characteristics are almost imperceptible. Various and multiple filters were applied to enhance the signal quality and its characteristics. For example, Kim et al. [49] converted the time-series signals into the frequency domain using Fourier transform, while some others [79,80,81] put a bandpass filter to extract the heartbeat signals and count the heart rate.

For studies that rely on the head movement method, they use almost identical processing steps. Both Li et al. [80] and Balakrishnan et al. [81] applied temporal filtering for the signal obtained from the head movement trajectories. Then they use principal component analysis (PCA) to obtain the periodic signal caused by the heartbeat. Finally, peak detection is used to help determine the heart rate.

Each study shows promising results by obtaining identical value compared to the reference ground truth, i.e., Kim et al. [49] obtained an average accuracy of 95.48%, Gault et al. [78] reached 90% accuracy, Li et al. [80] had a mean error of 2.7%, and finally, Balakrishnan et al. [81] achieve mean error of 1.5%. These results were achieved by comparing them to a contact-based device such as a pulse sensor and ECG. Unfortunately, there is no detailed information regarding the reference equipment model or specification.

### 3.3. Body Temperature

In most cases, the body temperature is measured using the LWIR type thermal camera. For example, Pan et al. [36]. used the P384-20 thermal camera, Rao et al. [37] used the Mobotix 16TR camera consisting of RGB and LWIR thermal camera, while Lewicki et al. [41] used the FLIR Lepton 3.5.

There are some steps to process the image and calculate the body temperature value. The process starts from obtaining the image from the thermal camera, followed by determining the ROI. In this case, some studies [37,41] used a combination of RGB and thermal cameras and needed a calibration process between two images. This RGB camera objective is to obtain enough information about the facial landmark and help select the ROI. Rao et al. [37] also implemented some advanced algorithms in their system, such as a prioritization algorithm to decide which person should be measured and background removal to separate person object and background environment. In terms of tracking, Rao et al. [37] used a neural network-based head tracker while another study [36] used an elliptical head tracking method.

Matching the two images from RGB cameras and thermal cameras also requires a series of processes. Instead of using Affine Transformation, Lewicki et al. [41] use coarse-grained field of view method, while Rao et al. [37] use manual offset approach and dynamic frame alignment.

Some authors also mentioned challenges related to accuracy. Rao et al. [37] implemented the temperature correction algorithm to compensate the temperature with the distance using a regression and multi-layer perceptron. The camera also needs to be calibrated in order to achieve a minimum error [82].

Each study has its procedure to get the body temperature value. For example, Pan et al. [36] determine the body temperature by measuring the highest temperature value from every point on the face. In comparison, Rao et al. [37] set some algorithms to prioritize the region of eyes and forehead followed by face and head. In contrast, Lewicki et al. [41] use the average value of each point on the face as the body temperature.

The eye’s inner canthus or medial canthus is known as the most accurate region of the face for measuring body temperature [83,84]. This region has a temperature that is almost identical to the eardrum temperature [85,86]. This region also has a similar temperature compared with the rectum, which has been the most identified as a reference for inner core body temperature [87]. However, because the selection of the inner eye canthus ROI in thermal images is quite challenging and requires an advanced method (for example, with machine learning or cross correlation [88]), several studies [87,89] have chosen the highest temperature value on the face or forehead as the body temperature which is also quite accurate.

Several methods were used to validate the system result by comparing it with the reference. Rao et al. [37], in their studies, used the black body measured temperature as the ground truth and obtained 100% sensitivity and 96.9% specificity from 105 people as the subject. On the other hand, Pan et al. [36] used an infrared ear thermometer as a reference and achieved a CAND value above 0.9.

## 4. Discussion

This systematic review provides an overview of studies that use thermal cameras to measure and monitor aspects of vital signs in the human body. This discussion section will present the advantages, disadvantages, challenges, and future trends and works.

### 4.1. Advantages of Thermal Camera-Based Physiological Measurement

A thermal camera can be used to measure several parameters such as RR, HR, and body temperature. Thus, it has the potential to be used in non-invasive, continuous measurements such as in neonatal ICU monitoring, long-term monitoring, fitness applications, and health screening. Moreover, during the COVID-19 pandemic, non-contact body temperature measurement methods promise hygiene. Mainly, it could be implemented as non-contact physiological measurement. Moreover, the thermal camera is able to operate in low light conditions. Therefore, using the thermal camera-based non-contact method offers more flexibility and convenience for the patient.

Since thermal camera overcomes the drawbacks of contact-based sensors, some clinical applications are using this method. For instance, it can provide continuous monitoring for neonates [4], classifying affective states [90], and monitoring exercise [91]. The thermal camera method is also effective for sleep monitoring [54] and estimating the movement during sleeping [5]. In addition, there are also some other implementations such as human thermal comfort modeling [92], lie detector [93,94], mood and stress-related disorders [95], sober and drunk classification [96], and many more.

### 4.2. Challenges of Thermal Camera-Based Physiological Measurement

A significant challenge of using a non-contact method that depends on the camera is that it is very susceptible to movement—both the motion of objects in the frame and the movement of the camera itself. Research in ROI tracking is needed to overcome this shortcoming. Additionally, separating partially obscured individuals or objects of the same temperature in thermal pictures can be challenging, as their pixels have the same intensity [97]. In these cases, including depth information or color edges can aid in disambiguation.

Related to the applicability, there is a concern for non-contact measurement using a thermal camera when faced with the current standard medical method in terms of accuracy and reliability. Although the accuracy of the prototype in this systematic review shows good results, it is not enough to exceed the performance of existing medical standard equipment. This happens because the systems developed in these studies have not been tested in actual health care conditions. Moreover, it has not been standardized or medically certified. Therefore, measurement involving several possibilities in real-case scenarios is an important direction to take. Several studies emphasize the importance of further clinical trials to ensure the reliability of the developed systems. The tests carried out need to combine several test scenarios to test the reliability of the system in measuring RR, for example, the use of various kinds of bed covers or blankets [54], the involvement of various patient demographics [40], and further clinical trials in the health institution such as hospital or clinic.

### 4.3. Future Trends and Works

Several suggestions and prospects need to be considered to improve the future of thermal cameras for physiological signal measurements.

#### 4.3.1. Healthcare Applications

In healthcare, the telemedicine revolution shifts illness prediction, prevention, and treatment from a hospital-centered reactive paradigm to a person-centered one. Driven by the COVID-19 pandemic and a growing need for home healthcare, telemedicine trends have the potential to transform and enhance healthcare delivery and accessibility. Non-contact physiological signal monitoring is a significant development that supports current telemedicine technology [98], including using thermal cameras as part of non-contact monitoring. Thermal cameras also provide a hygienic aspect for users where there are no components attached to the body to minimize contact between users.

For the popularization of health care, another critical challenge is related to low-cost implementation. Meanwhile, most of the studies in this systematic review have not explained development costs in detail. Research related to low-cost development is essential because the cost aspect is considered in implementing medical devices in developing countries [99,100].

#### 4.3.2. Machine Learning

Machine learning can be used as an enhancement to thermal images. As discussed in the previous section, a thermal camera generally has a small resolution. The resulting image can be enhanced to have a higher resolution by using the Thermal Image Enhancement method using convolutional neural network (TEN-CNN) [101]. Furthermore, the challenge in using machine learning is increasing the variety of datasets used to improve inference engine capabilities and accuracy. This is also expressed by Lyra et al. in their study [28]. The YOLOv4 they use relies on large-scale datasets to enhance their inference engine abilities. Improvements to this dataset also need to consider aspects of data variation for various races, ages, genders, weight and height, and other aspects.

#### 4.3.3. Multi-Parameter and Data Fusion

Multi-parameter measurement could be future work for the thermal camera-based physiological measurement system. Simplifying the measurement of several parameters will benefit patients using this system, especially multi-parameters on vital signs such as body temperature, blood pressure, heart rate, and respiratory rate. One study [102] that evaluated a multi-parameter wireless wearable sensor to monitor vital signs showed excellent and effective results. However, not many similar systems are accommodated in a non-invasive method.

Multi-parameter focuses on various parameters being measured. In contrast, data fusion is an aspect that combines several input data into the same process to achieve an output from a system. In this systematic review, there are several studies [40,42,47] that use data fusion in the form of combining several types of cameras. The aim and benefit of combining various sensors and data are to eliminate each sensor’ or method’s disadvantages and take advantage of every sensor. Further observation related to the combination of various sensors should be addressed to achieve better signal measurement.

## 5. Conclusions

In this paper, we have reviewed the existing literatures regarding thermal cameras to measure the human body’s respiratory rate, heart rate, and body temperature. The general process stages in processing thermal images for physiological signal measurements are discussed, compared, and evaluated to find advantages and challenges. The advantages of using a thermal camera in measuring physiological signals include comfort and convenience for the patient due to its non-invasive aspects, hygiene, and the ability to capture images in low light conditions. On the contrary, the challenges include how to reduce motion artifact and increase the accuracy and reliability of the physiological signal measurement system.

This systematic review contributes a comprehensive overview by providing highlights of current methodological concerns related to using the thermal camera to measure physiological signal. Furthermore, this systematic review can be used as an initial reference for researchers to identify the existing research gap. In addition, we also provide several future development directions, including integrating multi-parameter systems to improve functionality, using data fusion and machine learning technology to improve measurement accuracy and reliability, and developing low-cost thermal imaging applications to increase penetration.

## Figures and Tables

**Table 1:** Existing Systematic review.

Author	Published Year	Difference
Mikulska D. [19]	2006	Covered studies before 2006
Lahiri et al. [20]	2012	Published in 2012 and covered studies before 2012
El et al. [21]	2015	Only covered applications related to sports
Znamenskaya et al. [22]	2016	Limited to human psychophysiological conditions that are based on thermographic video
Zadeh et al. [1]	2016	Only covered breast cancer diagnostics by using thermal imaging
Moreira et al. [23]	2017	Developed checklist guidelines to assess skin temperature for sports and exercise medicine
Topalidou et al. [4]	2019	Database limited to EMBASE, MEDLINE, and MIDIRS and only covered thermal camera usage in neonatal care
Pan et al. [24]	2019	Focused on vein finder by using near infrared (NIR)
Aggarwal et al. [25]	2020	Focused on reviewing the accuracy of handheld thermal cameras
Foster et al. [26]	2021	Focused on assessing human core temperature using infrared thermometry
He et al. [27]	2021	Not focused on human vital signs

**Table 2:** List of Thermal Cameras Used in Some Studies in this Systematic Review Along with The Specifications Used.

Manufacturer	Model	Spectral Range	Temperature Accuracy	Thermal Sensitivity (NETD)	Maximum FPS and Resolutions	Used by
Flir Systems Inc., Wilsonville, OR, USA	Lepton 3.5	8 to 14 µm	±5 °C	<50 mK	8.7 FPS, 160 × 120 pixels	[40,41]
	A325	7 to 13.5 µm	±5 °C	<50 mK	60 FPS, 320 × 240 pixels	[42,43,44,45]
	Thermovision A40M	7 to 13.5 µm	±2 °C	<50 mK	60 FPS, 320 × 240 pixels	[46]
	A315	7.5 to 13 µm	±2 °C	<50 mK	60 FPS, 320 × 240 pixels	[47,48]
	P384-20	8 to 14 µm	±2 °C	<50 mK	50 FPS, 384 × 288 pixels	[36]
	T430sc	7.5 to 13 µm	±2 °C	<30 mK	12 FPS, 320 × 240 pixels	[49]
InfraTec GmbH, Dresden, Germany	VarioCAMR HD 820S	7.5 to 14 µm	±1 °C	<55 mK	30 FPS, 1024 × 768 pixels	[50]
Magnity Electronics Co., Ltd., Shanghai, China	MAG 62	7.5 to 14 µm	±2 °C	<60 mK	50 FPS, 640 × 480 pixels	[51,52,53]
Optris Gmbh, Berlin, Germany	Optris PI 450i	8 to 14 µm	±2 °C	<75 mK	80 FPS, 382 × 288 pixels	[28]
Seek Thermal Inc., Santa Barbara, CA, USA	Compact PRO	7.5 to 14 µm	-	<70 mK	>15 FPS, 320 × 240 pixels	[54]
Mobotix AG, Winnweiler, Germany	M16 TR	7.5 to 13 µm	±10 °C	<50 mK	9 FPS, 336 × 252 pixels	[37]

**Table 3:** Summary of Thermal Camera Usage Related to Respiratory.

Author	Objectives	Thermal Camera Model, FPS, and Dimension Used	Image and Signal Processing Tools	Algorithm Used	Validation Method	Performance
Chen et al. [52]	RR measurement	MAG 62, 10 FPS, 640 × 480 pixels	·Open CV: Image Processing Tools	·KLT: Coordinate Mapping ·RSQI_dtw: score each ROI	Compared with the GY-6620 sleep monitor	·Root Mean Square Error: 0.71 breaths/min and 0.76 breaths/min
Goldman et al. [46]	RR measurement	Thermovision A40, 50FPS, 320 × 240 pixels	·Matlab for signal processing software	·n/a	Compared with standard measurements of nasal pressure	·Intraclass correlation of 0.978 (0.991–0.954 95% CI)
Hu et al. [51]	RR measurement	MAG 62, 640 × 480 pixels	·All analysis conducted with Matlab R2014A	·Viola-Jones Algorithm for Cascade Object Detector ·Shi-Tomasi for the corner detection algorithm	Compared with human observers (manual counting)	·Accuracy for face, nose, and mouth: 98.46%, 95.38%, 84.62%
Hu, et al. [53]	RR and HR measurement	MAG 62, 30 FPS, 640 × 480 pixels	·Matlab R2014a for Image Processing	·Affine Transformation for transforming images	Compared with human observers (manual counting)	·Determination Coefficient: 0.831
Jagadev et al. [45]	RR measurement	Flir A325, 25 FPS, 320 × 240 pixels		·k-nearest neighbors (k-NN) Classifier ·the t-Stochastic Neighbor Embedding algorithm	Statistical calculation of sensitivity, precision, spurious cycle rate, missed cycle rate	·Sensitivity: 98.76% ·Precision: 99.07% ·Spurious cycle rate: 0.92% ·Missed cycle rate: 1.23%
Jagadev et al. [43]	RR measurement and classification	Flir A325, 25 FPS, 320 × 240 pixels		·Breath Detection algorithm for counting RR ·k-NN and SVM to classify the abnormalities	Statistical calculation of sensitivity, precision, spurious cycle rate, missed cycle rate	·Sensitivity: 97.2% ·Precision: 98.6% ·Spurious cycle rate: 1.4% ·Missed cycle rate: 2.8%
Jakkaew et al. [54]	RR measurement and body movement detection	Compact PRO, 17 FPS, 640 × 480 pixels	·minMaxLoc OpenCV: ROI Detection ·findContour: programming library to detect significant movement ·OpenCV: image processing framework		Compared with Go Direct respiratory belt	·Root Mean Square Error: 1.82 ± 0.75 bpm
Lyra et al. [28]	RR measurement	Optris PI 450i, 4 FPS, 382 × 288 pixels	·YOLO_mark: Labelling framework ·YOLOv4 with CSPDarknet53 Backbone: training framework ·YOLOv4-Tiny: Real-Time classifier framework		Compared with thoracic bioimpedance based patient monitor device (Philips, Amsterdam, The Netherlands)	·Intersection over unit (IoU): 0.70 ·IoU (tiny): 0.75 ·Mean Absolute Errors: 2.79 bpm, 2.69 bpm (Tiny)
Mutlu et al. [44]	RR measurement	Flir A325, 60 FPS, 320 × 240 pixels	·FLIR ResearchIRMax: Video Recording software ·Labview: camera trigger software ·MATLAB: analysis tools		Compared with a respiratory belt transducer containing a piezoelectric	·Median Error Rate: 6.2%
Negishi et al. [47]	RR measurement	Flir A315, 15 FPS, 320 × 240 pixels	·Labview: Image recording and analysis ·Grab cut: Extraction of contour ·Oriented FAST and Rotated Brief (ORB): feature matching ·dlib: ROI detection library ·OpenCV: Image Processing Tools		Compared with a respiratory effort belt (DL-231, S&ME, Japan)	·Root Mean Square Error: 2.52 RPM ·Correlation Coefficient 0.77
Negishi et al. [48]	RR and HR measurement	Flir A315, 15 FPS, 320 × 240 pixels	·Labview: Image recording and analysis ·Grab cut: Extraction of contour ·Oriented Fast and Rotated Brief: feature matching ·dlib: ROI detection library ·OpenCV: Image Processing Tools		Compared with a respiratory effort belt (DL-231, S&ME, Japan)	·Root Mean Square Error: 1.13 RPM ·Correlation Coefficient 0.92
Negishi et al. [42]	RR and HR measurement	Flir A325, 15 FPS, 320 × 240 pixels	·dlib: ROI detection library ·OpenCV: Image Processing Library	·Multiple signal classification (MUSIC) algorithm for signal estimation ·Homography Matrix for facial landmarking	Compared with a respiratory effort belt (DL-231, S&ME, Japan)	·Sensitivity: 85.7% ·Specificity: 90.1%
Pereira et al. [50]	RR measurement for infants	VarioCAMR HD 820S, 30 FPS, 1024 × 768 pixels	·Matlab 2017 for Evaluation and Signal Processing software		Compared with thoracic effort piezo plethysmography belt, namely SOMNOlab2	·Root Mean Square Error: (0.31 ± 0.09) breaths/min.
Scebba et al. [40]	RR measurement for apnea detection	NIR: See3cam_CU40 MV, 15 FPS, 336×190 pixels LWIR: Flir Lepton 3.5, 8.7 FPS, 160 × 120 pixels		·Smart Signal Quality Fusion (S2Fusion) for RR estimation ·Cascade Convolutional Neural Network (CCNN) for facial landmark ·KLT for tracking	Compared with piezo-resistive sensors based ezRIP module, Philips Respironics	· Median of Root Mean Square Error: 1.17 breaths/min

**Table 4:** List of Studies Involved Camera Fusion and Its Characteristic.

Authors	Fusion Camera Combination	Characteristic
Scebba et al. [40]	NIR and LWIR Camera	LWIR camera used for nostrils and chest ROI, NIR camera used for chest ROI
Negishi et al. [42,47,48]	RGB and LWIR Camera	RGB camera used for determining ROI and extracting PPG signals while LWIR camera used for extracting respiratory signal
Hu et al. [51]	RGB and LWIR Camera	RGB camera used for determining ROI while LWIR camera used for extracting respiratory signal
Chen et al. [52]	RGB and LWIR Camera	RGB camera used for determining ROI and alternative method to extract respiratory signal if no face detected while LWIR camera sued for extract respiratory signal if any face detected

## Data Availability

The data used in this review are from published primary studies available in the public domain.
